# Evaluation of the Stability of Newborn Hospital Parenteral Nutrition Solutions

**DOI:** 10.3390/pharmaceutics16030316

**Published:** 2024-02-23

**Authors:** Luis Otero-Millán, Brais Bea-Mascato, Jose Luis Legido Soto, Noemi Martínez-López-De-Castro, Natividad Lago-Rivero

**Affiliations:** 1 Pharmacy Department, University Hospital Complex of Vigo, 36312 Vigo, Spain; 2 NeumoVigo I+i Research Group, Galicia Sur Health Research Institute (IIS Galicia Sur), SERGAS-UVIGO, 36312 Vigo, Spain; 3 Innovation in Clinical Pharmacy Research Group (i-FARMA-Vigo), Galicia Sur Health Research Institute (IIS Galicia Sur), SERGAS-UVIGO, 36312 Vigo, Spain; 4 Applied Physic Department, Faculty of Sciences, University of Vigo, 36310 Vigo, Spain

**Keywords:** parenteral nutrition, physico-chemical stability, pediatric nutrition, critical care

## Abstract

(1) Background: parenteral nutrition (PN) solutions are an extremely complex mixture. It is composed of a multitude of chemical elements that can give rise to a large number of interactions that condition its stability and safety. The aim of this study was to evaluate the stability of PN solutions for preterm infants. (2) Methods: eight samples were prepared according to the protocol for prescribing PN in preterm infants. Samples PN1–PN7 had the normal progression of macronutrients and standard amounts of micronutrients for a 1 kg preterm infant. The PN8 sample had a high concentration of electrolytes, with the idea of forcing stability limits. Samples were stored both at room temperature and under refrigeration. Measurements of globule size, pH, density, and viscosity were performed in both storage protocols on different days after processing. (3) Results: the changes in the composition of the samples did not affect the evolution of the stability at the different measurement times and temperatures. Viscosity was affected by the compositional changes made in the PN samples, but no alterations due to time or temperature were observed. Density and pH remained stable, without significant changes due to time, storage temperature, or different composition. (4) Conclusion: all samples remained stable during the study period and did not undergo significant alterations due to compositional changes or different experimental conditions.

## 1. Introduction

Parenteral nutrition (PN) is a feeding technique that allows nutrients to be delivered directly into the bloodstream [1,2]. It is used in patients who are unable to meet their nutritional requirements via the enteral route, or in whom the gastrointestinal tract cannot be safely used. PN simultaneously provides macronutrients (amino acids, carbohydrates, and lipids), which constitute the caloric and protein intake [3], and micronutrients (electrolytes, vitamins, and trace elements), which complement the diet, preventing the development of deficits [4,5].

PN is used in many situations where the patient is incapable of oral or even enteral feeding. A particular case is that of premature infants. Premature birth radically suppresses the energy provided by placental transport, posing a nutritional emergency for the newborn. This is because their digestive system is unable to perform its functions, i.e., to handle and absorb the energy necessary for extrauterine life. A large majority of preterm infants, especially those with a gestational age of less than 32 weeks and others who are older but small for their age, are unable to meet their nutritional requirements using the enteral route. The fundamental objective of this technique is to avoid early malnutrition in order to reduce morbidity and mortality [6].

PN should be administered to all infants weighing <1500 g during the first days of life (at least until 75% enteral tolerance is achieved) or in heavier infants where enteral intake is restricted or contraindicated. In children in whom it is indicated, it should be started within the first day of life (as soon as venous access is available and the patient is stabilized). In this way, the calculated nutritional requirements of the preterm infant will be reached as soon as possible. Rapid initiation of PN is crucial to minimize losses and improve growth rates [7]. The body composition of a newborn weighing less than 1 kg contains 1% fat and 8% protein, which represents a non-protein caloric reserve of 110 Kcal/kg, too little to maintain basal needs during the first four days of life. If we add a situation of clinical instability, such as respiratory failure or sepsis/shock situations, common in these patients, the metabolic consumption is greater, and the nutritional urgency is aggravated [8]. It is worth noting the difference in ratio with adults, where the basic requirements are usually between 25 Kcal/kg and 1.5 g protein/day (calorie:nitrogen ratio 80–100:1) while in neonates it is about 120 Kcal/kg and 3 g protein/day (ratio 225–250:1). Another important difference is the amount of essential electrolytes or micronutrients added. The recommended daily amounts for adults and children are different. For example, the recommended amount of calcium for adults is in the range of 0.03–0.2 mMol/kg/day, while for pediatric patients it is between 0.5–2 mMol/kg/day [9].

When we initiate PN we are dealing with tremendously complex mixtures. They are lipid emulsions with several components in solution: amino acids, phosphate salts, calcium, magnesium, sodium, potassium, vitamins, and trace elements (copper, zinc, manganese, etc.). For that reason, the physicochemical processes that can occur are very diverse and unpredictable [10], hence the difficulty in unequivocally determining their stability and safety [11]. One of the main physicochemical stability problems is the destabilization of the lipid emulsion. Factors such as high cation concentration, low amino acid concentrations, or sudden changes in pH can influence globular aggregation, leading to coalescence and phase separation, or the formation of precipitates. In addition, degradation processes of components, such as vitamins, or the formation of toxic species, such as peroxides, can occur in these samples. These processes are related to chemical stability aspects [12]. For premature infants, it is necessary to prepare PN with high concentrations of glucose, lipids, amino acids, sodium, potassium, magnesium, and especially calcium and phosphorus to maintain proper development. Their preparation is often limited by the lack of stability standards for the different components of the solution. This causes the clinician to prioritize the possible occurrence of instability in the solution over the nutritional needs of the patient. There is some uncertainty as to the stability limits of the mixture depending on its composition [13]. The higher the amount of calcium that must be added to neonatal PN, the more likely that stability problems will emerge [14,15]. Therefore, it is still common practice in some centers to infuse lipid emulsions separately from the rest of the PN components due to the stability problems caused by the higher amount of calcium and lower concentration of amino acids [16,17].

Administration of unstable parenteral nutrition, in which precipitates have formed or fat globules have been added, may compromise patient outcomes, increasing morbidity and mortality [18,19]. For this reason, it is vitally important to determine the ranges within which the nutrient concentration could be adjusted without affecting the overall stability of the solution [20].

In this study, we performed a comprehensive evaluation of the stability of different newborn PN, considering changes in composition and storage protocol.

## 2. Materials and Methods

### 2.1. Sample Preparation and Storage

This study consists of the evaluation of eight pediatric parenteral nutrition solutions (Table 1). The solutions were prepared following the protocol for prescribing PN in premature infants of the Hospital Universitario de Vigo, adjusting the calculation to the requirements of a premature newborn weighing 1 kg. The processing procedure followed the standards and procedures concerning the cleaning and disinfection of the area, the use of aseptic techniques, the use of laminar flow cabinets (LFC), and the evaluation of the finished product. The latest Spanish consensus on the preparation of PN mixtures, drawn up by the Working Group on Artificial Nutrition Pharmacy of the Spanish Society of Parenteral and Enteral Nutrition (SENPE) in 2008 [2], was followed for the preparation. Amino acids and glucose shall be introduced into the NP bag first. Glucose and lipids shall never be mixed directly without the presence of amino acids. Monovalent electrolytes (Na and K) shall be added next, followed by phosphate and magnesium. Lastly, trace elements such as calcium are added, as far away as possible from phosphate to avoid the phenomenon of localized concentration that increases the risk of precipitation. The lipid emulsion is incorporated into the mixture of amino acids, glucose, electrolytes, and trace elements. In this way, the visual inspection of the mixture is facilitated. Finally, the vitamins are added to the samples.

From sample 1 to sample 7 (PN1–PN7), the nutrients progressed as performed in real clinical practice during the first days of life. In the last sample (PN8), a critical composition for stability was sought. To this end, electrolyte concentrations were increased by 50–100%, with respect to the maximum amount recommended by protocol. Macronutrient concentrations were maintained at basal levels. In the case of lipids, the concentration was further increased in line with the protocol. To begin, 100 mL per sample was prepared and the amounts and concentrations of all components were progressed according to the center’s protocol for parenteral nutrition in preterm infants [21]. On day 0, a single stock sample was prepared, from which two aliquots of 50 mL were separated and stored at room temperature (RT) or in a refrigerator (4 °C). The sample intended for storage at room temperature was kept in the original bag (Ethyl Vinyl Acetate (EVA) plastic), free of air and sealed. On each day of analysis, the amount needed for the different analyses was extracted. The quantity set aside for refrigeration was dosed into sterile, air-free syringes (polypropylene) with a Luer lock cap. For the measurements, the amount needed for the analyses was withdrawn from the syringe. The samples were kept protected from light and free of air at all times. To keep the samples free of microbiological contamination, sterile material was always used. Furthermore, the preparation was carried out in an aseptic environment in laminar flow cabinets.

### 2.2. Evaluation of Globule Size

The stability of the lipid emulsion shall be analyzed by using a Beckman Coulter LS I3 320 (Barcelona, Spain), obtaining the mean globule size and size distribution of the emulsion. This device uses the laser diffraction (DL) technique and angular analysis to determine particle size. It emits a laser beam towards the sample and measures the scattering of light at different angles. Specifically, this instrument uses the DL plus, which is an advanced polarization intensity differential scattering (PIDS) technology that enables high-resolution measurement. The measuring range is between 10 nm–3500 µm.

Globule size measurements were performed on days 0, 1, 3, and 7, both in samples kept at room temperature and those at 4 °C. On each measurement day, aliquots with an excess volume were extracted into several Eppendorf tubes from the original sample. On each measurement day, they were sent to the external laboratory manager for analysis. All measurements were performed according to standard laboratory practice and the specific instructions of the equipment manufacturer.

### 2.3. Density and Viscosity

Viscosity measurements were carried out on days 0, 1, 3, and 7, both on samples kept at RT and 4 °C under temperature control at 25 °C. Each measurement is the result of the average of six determinations for viscosity. In the case of density, we took one measurement on day 0. The dynamic viscosity was calculated by multiplying the kinematic viscosity by the corresponding density.

The density measurements were carried out with an Anton Paar DMA4500 vibrating tube mechanical oscillation density meter (Graz, Austria). The temperature of the density meter is set by two PT100 probes integrated in the device itself and the calibration of the system was carried out using standard fluids [22,23].

For viscosity determination, we use an Anton Paar AMV 200 viscometer (Graz, Austria). The test temperature is kept constant by means of a PolyScience circulation bath (Cham, Suiza). Calibration is carried out using standard liquids for each of the capillaries [23,24].

### 2.4. PH Assessment

The pH of the mixtures studied was measured with a Crison pH meter model Basic 20+ (Barcelona, Spain) [25]. pH measurements were carried out on days 0, 1, 3, and 7, both on samples stored at room temperature and 4 °C. Measurements were performed under temperature control at 25 °C. In total, 2.5 mL of each sample was placed in 15 mL polypropylene centrifuge tubes. Once the tubes were filled, they were placed on a rack in a thermostatic bath to reach the set temperature of 25 °C. We took one measurement on each day.

### 2.5. Visual Controls

After preparation and on each day of analysis, the bags were macroscopically examined for phase separation, particle appearance, or color changes of the solution.

### 2.6. Statistical Analysis

In the globule size analysis, the data were presented using the median with the standard deviation. In the case of the rest of the experiments, the graphs represent the mean of the different replicates performed in the study (where replicates were available).

Analyses were performed to assess changes in globule size distribution due to the different composition calculated for each sample and due to the different storage temperature. With this analysis, the influence of time, temperature, and composition on stability was evaluated. First, to analyze the influence of composition, the comparison was made between sample PN1 and the rest of the PNx samples on the same day of measurement and under the same storage conditions (e.g., PN1 at day 7 in ambient versus PN5 at day 7 in ambient). The PN1 sample was used as the reference from which to make comparisons, as it was considered the most appropriate due to its low concentration in all its components and theoretically greater stability. The PN1 sample is closest to clinical use, has lower concentrations of cations, and contains concentrations of macro-nutrients commonly used in clinical practice. Comparisons between different time and temperature points in each sample were performed using the non-parametric Friedman test for ranked values. Values of *p* < 0.05 were considered statistically significant. This test was used to analyze the evolution of globule size in each sample and temperature over time.

Further analyses compared the differences due to the different composition of the samples in each time point and storage temperature (groups). A Kruskal–Wallis test was performed in order to analyze the variance between the different groups. In cases where this test yielded positive results (*p*-value < 0.05), a post-hoc analysis was continued using a pairwise comparison with the Wilcoxon test. A Benja–mini–Hochberg multi-test correction (BH/FDR) was applied to the *p*-values of the Wilcoxon test and an adjusted *p*-value (*p*.adj) of less than 0.05 was considered significant. These values are indicated in the figures with “*”: *: *p*.adj < 0.05; **: *p*.adj < 0.001; ***: *p*.adj < 0.0001; ****: *p*.adj < 0.00001. Moreover, to analyze the influence of the storage temperature on globule size, each sample at RT was compared with its corresponding sample at 4 °C.

For statistical analyses and visualization, R (v4.2.2)^®^, Rstudio^®^ (v4.9.4), and the R packages ggplot2 (v 3.4.2)^®^, ggpubr (v0.6.0)^®^, tidyverse (v2.0.0)^®^, and rstatix (v0.7.2)^®^ were used. Due to the low number of samples in certain experiments and the fact that the distribution of the data in the globule sizing experiments did not follow a normal distribution, non-parametric statistics were used.

## 3. Results

### 3.1. Globule Size Remains Stable during the Study Period

Day 0 corresponds to the day of sample preparation. Only one sample was prepared, from which aliquots were separated to be stored at room temperature and in the refrigerator for the analyses on the different days. The data corresponding to day 0 are interpreted as 4 °C data.

**Figure 1:**
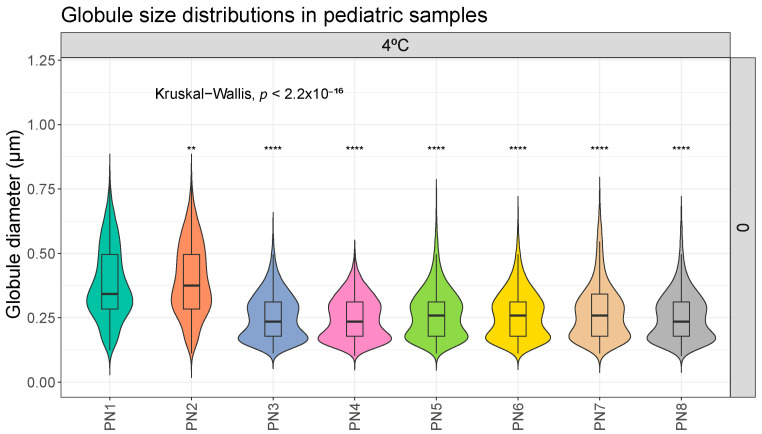
Distribution of globule size (µm) in different pediatric parenteral nutrition (PN) samples on day 0, at 4 °C. **: *p*.adj < 0.001; ****: *p*.adj < 0.00001.

In the analysis carried out to compare the differences due to the different composition, sample PN1 shows statistically significant differences with respect to the rest of the samples. Figure 1 shows this difference in its basal measurements on day 0. This tendency remains constant during all the conditions studied (on all the days of analysis and both at room temperature and in the refrigerator). The complete comparison, made according to the different days of analysis and storage conditions, can be found in Supplementary Materials (Figures S1 and S2).

**Figure 2:**
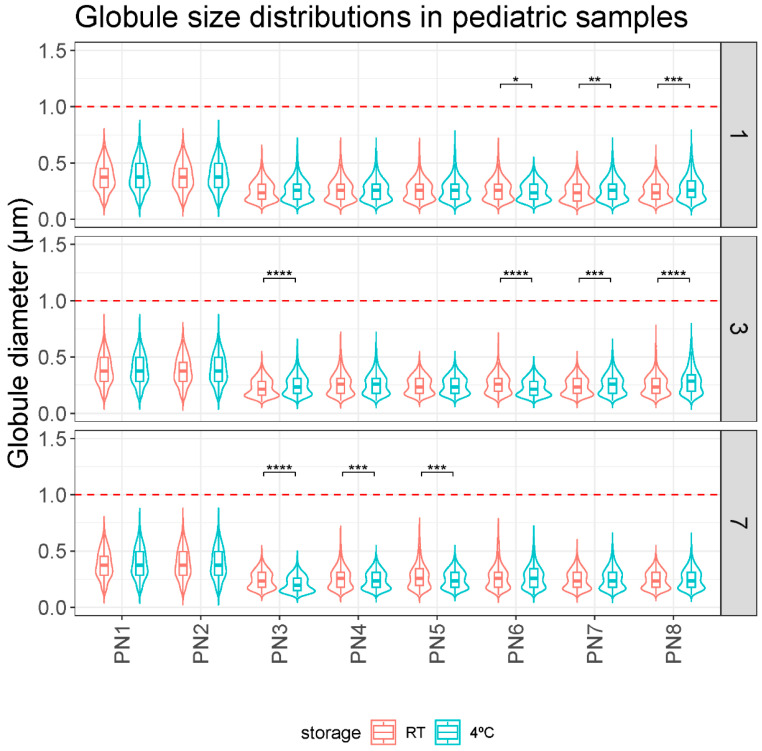
Comparison of globule size distributions (µm) between storage protocols (RT and 4 °C) over time (days 1, 3, and 7). *: *p*.adj < 0.05; **: *p*.adj < 0.001; ***: *p*.adj < 0.0001; ****: *p*.adj < 0.00001.

On the other hand, Figure 2 shows the analysis performed to evaluate the impact of storage temperature. Statistically significant differences were observed between some samples.

Overall, no relevant differences were observed in the globule size of the PN1–PN8 samples according to their different storage protocol on any day of analysis. The mean globule size was always less than 0.5 microns and no globule fraction larger than 1 micron was detected in any measurement (Table 2).

### 3.2. The Viscosity of Parenteral Nutrition Solutions Varies as Their Composition Is Altered

To analyze the viscosity of our mixtures, measurements were made on the different days (0, 1, 3, and 7) on samples kept in ambient and refrigerated conditions. Each measurement is the result of the average of six determinations for viscosity. The dynamic viscosity was calculated with the density measurements taken on day 0.

**Figure 3:**
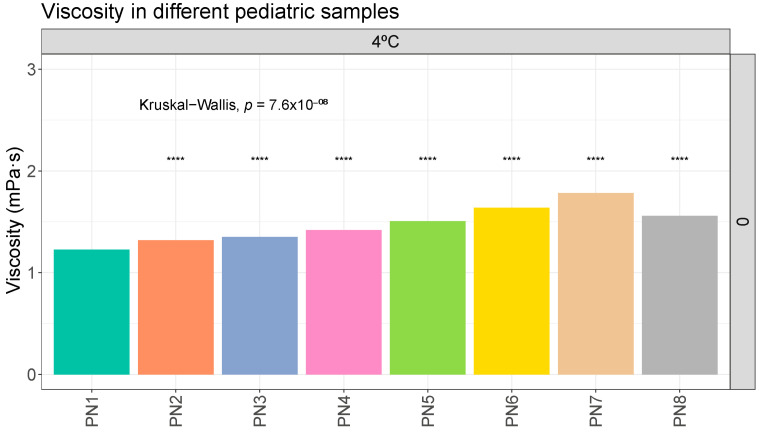
Viscosity (mPa·s) evolution in different pediatric parenteral nutrition solutions on day 0 at 4 °C. ****: *p*.adj < 0.00001.

A statistical analysis like the one carried out with the globule size data was performed, where the viscosity data of sample PN1 was compared with the rest of the PNx samples at the same storage times and conditions. Significant differences were observed between the viscosity results of sample PN1 and the other samples due to the different composition (Figure 3). The same trend was observed on the remaining days of analysis and under the different storage conditions. The complete data are shown in the Supplementary Materials (Figures S1 and S2).

In general, no relevant variations in viscosity values were observed due to time or storage protocol (Table 3).

### 3.3. Density of Parenteral Nutrition Solutions Is Not Altered by Nutrient Composition

The evaluation of the density in the different solutions showed no significant differences in the evolution of the samples (Table 4).

### 3.4. Variations in the Nutrient Content of the Additive Solutions Do Not Change the pH

Slight variations in pH values were observed between the different samples over time and under the different storage protocols. The average pH value (mean among all results obtained) was 6.53 ± 0.13 (6.28–6.84) (Table 5).

## 4. Discussion

This study evaluated the stability of different pediatric PN solutions. In the first case, the globular size results showed the stability of the tested PNs (Figure 1 and Figure 2). Based on these results, storage of the samples at room temperature for 7 days did not pose any problems in terms of stability. Although clinical practice suggests a stability limit of 5 days at 4 °C, this appears to be considerably higher. Since we did not detect instability features at 7 days in any sample, larger studies would be needed to define the critical point. Some statistically significant differences were observed in the room temperature versus fridge comparison but these differences do not follow any clear trend (Figure 2). Thus, they would appear to be mainly due to random events or variability of the measurement technique. They would therefore have no clinical relevance.

The essential size characteristics of a lipid emulsion include the mean droplet diameter (MDD) and the range of various droplet diameters distributed around the mean diameter. In particular, the amount of fat globules comprising the large-diameter tail of the globule size distribution is especially important with respect to infusion safety (>5 µm). The United States Pharmacopeia (USP) in Chapter 729 [26] establishes the methods that are used for the determination of MDD and the distribution of large-diameter globule sizes in lipid emulsions. Additionally, these two regions of the globule size distribution must be controlled within specified limits: Method I: 0.5 microns as the upper limit of average droplet size of the emulsion, and Method II: the size distribution of the emulsion is evaluated. The percentage of the weighted volume of fat residing in a droplet of diameter > 5 µm in the scattered phase (PFAT5) should be less than 0.05%. According to our data, these control parameters, proposed by the USP, are met. In our samples, no globules > 5 µm were detected in any sample. Also, no globular fraction larger than 1 µm was detected (Figure 1 and Figure 2).

This is also observed in other similar studies on PN for preterm infants. In the study by Skouroliakou et al. [27], the physicochemical stability of PN in neonates was analyzed. Regarding the globule size results, the analyses showed no globules larger than 4 µm; moreover, 99% of the size distribution was found to be smaller than 1 µm. Their study protocol was very similar to ours. Their samples were stored at room temperature and at 4 °C and analyzed on study days 0, 1, 3, 7. The equipment used for this globule size analysis was a Mastersizer X (Malvern Instrument Ltd., Malvern, UK) using DL. Therefore, according to their results, they observed that the compositions studied were physically stable during the study conditions.

The study by Watrobska-Swietlikowska et al. [28] aimed to assess the stability of neonatal PN mixtures. Globule size was analyzed by DL and light microscopy (OM) and photon correlation spectroscopy, pH, Z-potential, and surface tension. These analyses were performed on day 0 and day 1 after storage at room temperature. Additionally, analyses were performed at 21 days of storage in two-chamber bags. The lipid emulsion was kept separate from the rest of the components until mixing. On day 21, all the components were mixed, and the variables were analyzed at 0 and 24 h at room temperature. The results showed a size distribution always smaller than 1 µm, regardless of the technique used. Only two problematic compositions were observed, with a small percentage of globules of about 5 µm after 21 days of storage. The different techniques used in this study obtained similar results, always with size distributions below 1 µm. This study did not show complete size distributions and did not explain the differences obtained in the two samples where larger globules were observed. However, their results are consistent with ours in terms of size distributions, even though they did not follow the same protocol. A second study by Watrobska-Swietlikowska et al. [29] used a similar study protocol, based on DL analysis and photon correlation spectroscopy. Again, no globules larger than 1 µm were observed. Therefore, our results are consistent with the literature.

For all measurements, the specifications provided by the equipment manufacturers have been strictly followed, but it could be that the failure to detect fractions of globules larger than 1 µm may be due to technical limitations of the equipment or the measurement method. When analyzing emulsions with a high concentration of small globules relative to the larger globules, a “fog effect” can occur. The high concentration of small particles in front of the beam means that a large part of the radiation is reflected back towards the source and does not reach the detector. This causes errors and distortions in the larger particle populations, i.e., those of larger globular size. However, our results are congruent with the literature, where data for large globule fractions are also not reported for samples of similar composition but measured using other methodologies.

Other possible sources of error would be the adsorption of materials to the containers used for sample storage and measurement. Gonyon et al. [30] aimed to analyze the relationship between changes in emulsion globule size distribution and adsorption to the container of lipid emulsions in PN mixtures. For this purpose, samples stored in glass bottles and in Ethyl Vinyl Acetate (EVA) plastic were compared. Globule size distributions and the amount of lipids in the containers were measured by liquid chromatography and gravimetric changes in the containers. The results showed clearly higher adsorption to plastic containers than to glass containers according to all measurements. PFAT5 for samples in EVA containers showed a 75% reduction compared to a marginal decrease of PFAT5 in the glass container. Chromatographic extraction and measurement of the containers showed that the amount of lipids associated with the EVA surfaces increased steadily with emulsion exposure time, while the glass showed a significantly lower lipid content. Gravimetric measurements confirmed that the EVA containers gained significant mass during storage. Our samples were prepared in EVA bags. Thus, this is a possible cause that we did not find >1 µm globule fractions or elevated PFAT in our analyses. However, to confirm this it would be necessary to plan a new study where each sample is stored in different containers and each container is analyzed.

Other studies also state that different factors, such as agitation and the introduction of air into the bags, could interfere with globule size measurements. Driscoll et al. [31,32] attribute a higher initial PFAT5 to a higher amount of air or bubbles in the first measurements, which disappear after storage. In our study we cannot confirm whether this phenomenon had an impact on our results, as we have not made any observations or measurements in this respect.

Finally, a different scenario from the above would be that excessive agitation could break up aggregates of globules or split large globules. Theoretically, coalescence, the formation of large globules, is an irreversible process, so it does not seem a likely hypothesis. However, the measurement of aggregates as large globules could be a possible explanation. The aggregates would disintegrate in later measurements if the sample were subjected to adequate agitation.

Our statistical analysis showed significant differences in composition, in particular the MDD of samples PN1 and PN2 and storage protocol (Figure 1). Samples PN1 and PN2 had a larger globule size than the other samples. It would be important to distinguish whether these differences have clinical relevance. This change means going from a MDD of 0.379–0.397 µm in the PN1–PN2 samples to 0.207–0.285 µm in the other samples (minimum and maximum values of all measurements taken at different times and different storage conditions). The differences found in the ambient versus fridge comparisons are even smaller. For example, sample PN5 (RT and D7) has a MDD of 0.274 µm compared with 0.249 µm in the PN5 (4 °C and D7) sample. All values, as mentioned above, are within the USP parameter of MDD < 5 µm, so, in principle, these differences should not have a major impact on stability [26].

In the results obtained for density, viscosity, and pH, the variations observed at different storage times and temperatures were practically negligible. No trends towards instability were observed in these variables (Table 3, Table 4 and Table 5). Although samples PN1 and PN2 showed different behaviors in terms of globule size distribution, no differences were observed for the other properties analyzed. These properties are likely to be altered only in scenarios with a very marked destabilization of the sample, such as phase separation. In this aspect, it could be interesting to carry out this type of measurement to see if they deviate from the reference values, which would mean a significant destabilization. Only a slight increase in the viscosities of the solutions was observed, which could be correlated with the increase in the concentration of macronutrients. The literature does not provide critical reference values for the density and viscosity variables. There are no defined values for the pH variable either. However, the literature indicates that extremes of pH (pH > 8 or pH < 4.5) could be critical for the stability of the product.

In stability studies, it is common to measure other properties of emulsions, such as the zeta potential. A zeta potential below −20 mV increases the attraction derived from Van der Waals forces over electrostatic repulsion [10,20]. This would lead to destabilization of the emulsion. The acidification of the pH causes a decrease in the zeta potential due to the reduction of the negative charge of the phospholipids. This could explain the larger MDD size in PN1/2 samples. However, further studies measuring the zeta potential are needed to confirm these hypotheses.

In our study, CAN value varies from 750 to 2370 mMol/L between PN1 and PN8, respectively. We did not find a relationship between a higher CAN value and a higher MDD, increases in large cell fraction or a higher occurrence of precipitate. In other words, no relationship was observed between a higher CAN value and the instability of PN mixtures. Although the CAN calculation is simple, it does not consider other factors such as pH and amino acid concentration, which play an important role in stability [33]. Not taking other factors into account can lead to an erroneous estimation of sample stability. For this reason, the use and importance of CAN as a prognostic value for stability in NP formulations has decreased over the years. Currently, the USP recommends other parameters such as MDD or PFAT5. These parameters have been shown to have a direct relationship with lipid coalescence and aggregation and therefore with the instability of these mixtures. However, in daily work, immediate calculations of parameters such as MDD are not readily available. For this reason, CAN, calcium, phosphorus, or macronutrient concentrations are still used in many clinical centers as a guide parameter to predict aggregation and instability states. In this context, more studies of this type are needed to establish efficient and rapid control parameters that can be applied to routine clinical practice.

## 5. Conclusions

In conclusion, all samples showed stability during the study period. No changes in globule size were detected due to the different storage protocol and USP parameters were met in all samples under different conditions. Density and viscosity remain relatively constant in the parenteral nutrition samples, but slight increases are observed with increasing macronutrient concentrations. The pH remains stable within acceptable ranges throughout the study period. Elevated cation concentrations (elevated CAN) do not correlate with increased lipid emulsion instability. It should be noted that sample handling conditions for globule size analysis may interfere with the interpretation of results, such as excessive shaking or containers used for transport.

We should conduct studies covering the maximum number of variables and with the products used today, to provide new and updated information about PN stability. In order to extrapolate information and increase knowledge about emulsion stability, more complete and comparable studies should be carried out.

## Figures and Tables

**Table 1:** Composition of the pediatric samples used in the study with their respective CAN and OSM values.

Sample	N (g/L)	Prot (g/L)	Gluc (g/L)	Lip (g/L)	Na (mMol/L)	K (mMol/L)	Mg (mMol/L)	Ca (mMol/L)	P (mMol/L)	OSM (mOsm/L)	CAN (mMol/L)
PN1	3.14	19.64	71.43	7.14	20.00	10.00	1.25	10.00	10.00	743.33	750
PN2	3.38	21.10	80.25	11.38	30.00	15.00	2.25	15.00	15.00	878.62	1149
PN3	3.56	22.22	87.11	14.78	35.00	20.00	2.60	17.50	17.50	969.77	1341
PN4	3.70	23.13	92.60	17.50	40.00	30.00	3.00	20.00	20.00	1054.23	1542
PN5	4.20	26.25	106.80	21.60	40.00	30.00	3.00	20.00	20.00	1178.95	1542
PN6	4.70	29.38	121.00	25.80	50.00	35.00	3.50	22.50	25.00	1350.08	1749
PN7	5.20	32.50	135.20	29.90	60.00	40.00	4.00	25.00	30.00	1520.80	1956
PN8	3.14	19.64	71.43	40.00	80.00	50.00	5.00	30.00	40.00	1150.91	2370

PN1–PN8: Pediatric samples; N: nitrogen; Prot: protein (estimated using conversion factor 6.25 g Protein/g nitrogen; commercial source used Aminoven Infant 10% Fresenius Kabi^®^ Barcelona, Spain); Gluc: glucose (Glucose 50% Grifols^®^ Barcelona, Spain); Lip: lipids (Lipoplus 20% Braun^®^ Melsungen, Germany); OSM: osmolarity; CAN: critical aggregation number, calculated according to cation concentration to analyse its relationship to stability (CAN = a + 64 b + 729 c; where a, b and c are the sum of the concentrations (mmol/L) of mono-, di- and trivalent cations, respectively). Other components used: Sodium chloride 20% Braun^®^, Potassium acetate 1 M Braun^®^, Sodium glycerophosphate: Glycophos Fresenius Kabi^®^, Calcium gluconate: Suplecal Braun^®^, Magnesium sulfate 15% Genfarma^®^ Madrid, Spain, Vitamins: Vitalipid Fresenius Kabi^®^, Trace elements: Meinsol Oligo-zinc Fresenius Kabi^®^, Water for injection Grifols^®^.

**Table 2:** MDD (µm) and standard deviation of pediatric samples PN1–PN8. All days of analysis (0, 1, 3, and 7) and both storage conditions (RT: Room temperature, 4 °C: refrigerator).

Sample	Day 0	Day 1		Day 3		Day 7	
	4 °C	RT	4 °C	RT	4 °C	RT	4 °C
PN1	0.382 ± 0.146	0.379 ± 0.128	0.388 ± 0.137	0.384 ± 0.134	0.389 ± 0.133	0.382 ± 0.130	0.390 ± 0.136
PN2	0.397 ± 0.146	0.382 ± 0.128	0.387 ± 0.134	0.381 ± 0.130	0.389 ± 0.133	0.388 ± 0.137	0.389 ± 0.139
PN3	0.255 ± 0.094	0.254 ± 0.092	0.258 ± 0.097	0.229 ± 0.081	0.252 ± 0.092	0.245 ± 0.081	0.207 ± 0.074
PN4	0.247 ± 0.086	0.260 ± 0.101	0.261 ± 0.098	0.260 ± 0.100	0.261 ± 0.097	0.262 ± 0.099	0.241 ± 0.081
PN5	0.267 ± 0.101	0.261 ± 0.095	0.265 ± 0.100	0.249 ± 0.081	0.249 ± 0.080	0.274 ± 0.111	0.249 ± 0.082
PN6	0.262 ± 0.098	0.264 ± 0.099	0.250 ± 0.085	0.262 ± 0.091	0.230 ± 0.077	0.270 ± 0.112	0.267 ± 0.102
PN7	0.273 ± 0.115	0.246 ± 0.092	0.261 ± 0.098	0.240 ± 0.081	0.256 ± 0.093	0.245 ± 0.086	0.254 ± 0.095
PN8	0.257 ± 0.099	0.254 ± 0.091	0.275 ± 0.110	0.253 ± 0.092	0.285 ± 0.117	0.243 ± 0.081	0.253 ± 0.091

**Table 3:** Viscosity (mPa·s) evolution and standard deviation in different pediatric parenteral nutrition solutions on different days (0, 1, 3, 7) and temperatures (RT, 4 °C). RT: room temperature.

Sample	Day 0	Day 1		Day 3		Day 7	
	4 °C	RT	4 °C	RT	4 °C	RT	4 °C
PN1	1.264 ± 0.004	1.267 ± 0.007	1.261 ± 0.006	1.261 ± 0.006	1.280 ± 0.040	1.256 ± 0.003	1.261 ± 0.006
PN2	1.367 ± 0.004	1.343 ± 0.007	1.370 ± 0.004	1.349 ± 0.004	1.350 ± 0.006	1.347 ± 0.008	1.354 ± 0.004
PN3	1.400 ± 0.013	1.405 ± 0.005	1.413 ± 0.005	1.418 ± 0.003	1.407 ± 0.005	1.413 ± 0.004	1.415 ± 0.007
PN4	1.476 ± 0.007	1.455 ± 0.003	1.471 ± 0.004	1.471 ± 0.006	1.463 ± 0.004	1.482 ± 0.007	1.487 ± 0.006
PN5	1.580 ± 0.007	1.589 ± 0.005	1.582 ± 0.005	1.584 ± 0.005	1.584 ± 0.005	1.603 ± 0.005	1.591 ± 0.004
PN6	1.733 ± 0.004	1.743 ± 0.003	1.743 ± 0.003	1.740 ± 0.007	1.738 ± 0.004	1.743 ± 0.004	1.744 ± 0.003
PN7	1.904 ± 0.004	1.900 ± 0.005	1.908 ± 0.004	2.091 ± 0.006	2.092 ± 0.006	1.899 ± 0.005	1.893 ± 0.006
PN8	1.554 ± 0.005	1.579 ± 0.006	1.524 ± 0.005	1.526 ± 0.004	1.526 ± 0.004	1.541 ± 0.006	1.530 ± 0.008

**Table 4:** Density (g/cm^3^) evaluation in different pediatric parenteral nutrition solutions.

Sample	Density Day 0 (4 °C)
PN1	1.035
PN2	1.041
PN3	1.045
PN4	1.047
PN5	1.055
PN6	1.062
PN7	1.069
PN8	1.052

**Table 5:** Ph evaluation in different pediatric parenteral nutrition solutions on different days (0, 1, 3, 7) and at temperatures (RT, 4 °C). RT: room temperature.

Sample	Day 0	Day 1		Day 3		Day 7	
	4 °C	RT	4 °C	RT	4 °C	RT	4 °C
PN1	6.28	6.41	6.4	6.44	6.44	6.48	6.55
PN2	6.46	6.43	6.48	6.51	6.52	6.55	6.58
PN3	6.68	6.70	6.62	6.55	6.52	6.48	6.47
PN4	6.69	6.66	6.63	6.50	6.54	6.48	6.49
PN5	6.40	6.42	6.42	6.42	6.43	6.40	6.46
PN6	6.45	6.44	6.47	6.43	6.44	6.49	6.51
PN7	6.56	6.48	6.40	6.51	6.58	6.53	6.58
PN8	6.78	6.77	6.80	6.76	6.79	6.82	6.84

## Data Availability

The data presented in this study are available in this article (and Supplementary Materials).

## Supplementary Materials

The following supporting information can be downloaded at: https://www.mdpi.com/article/10.3390/pharmaceutics16030316/s1, Figure S1. Representation of how the mean globule size evolves over the study time (0, 1, 3 and 7 days) for each sample and storage protocol (RT: Room temperature, 4 °C: refrigerator); Figure S2. Distribution of globule size (µm) in different pediatric parenteral nutrition (PN) solutions on different days (0, 1, 3, 7) and temperatures (RT, 4 °C). RT: room temperature; Figure S3. Viscosity (mPa·s) evolution in different pediatric parenteral nutrition solutions on different days (0, 1, 3, 7) and temperatures (RT, 4 °C). RT: room temperature.
