# Supplementary material for: Evaluation of the Stability of Newborn Hospital Parenteral Nutrition Solutions
Source: Pharmaceutics. 2024 Feb 23;16(3):316. doi: 10.3390/pharmaceutics16030316 (PMC10975575; doi:10.3390/pharmaceutics16030316)
Supplement: Supplementary file 1 [file pharmaceutics-16-00316-s001.zip › pharmaceutics-2813562-supplementary.pdf]

Supplementary Materials

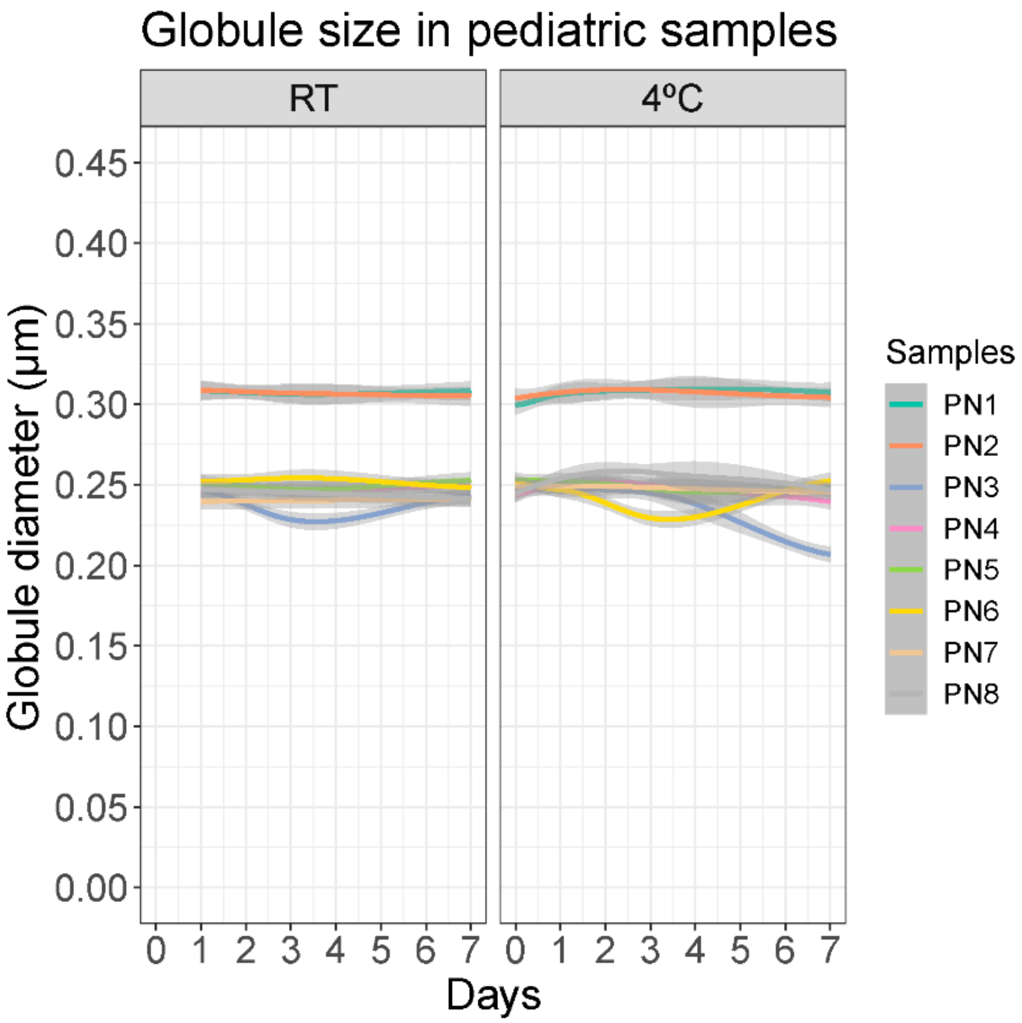

Figure S1. Representation of how the mean globule size evolves over the study time (0, 1, 3 and 7 days) for each sample and storage protocol (RT: Room temperature, 4ºC: refrigerator).

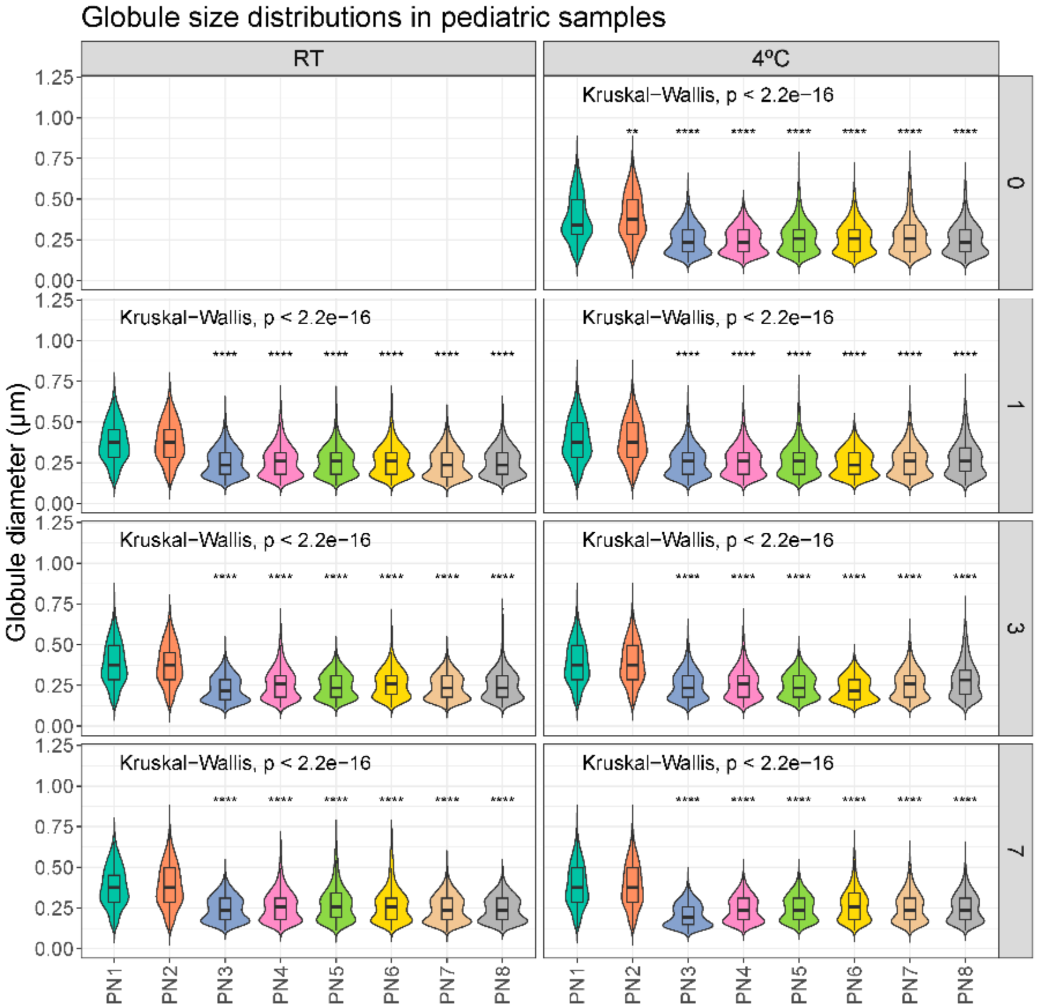

Figure S2. Distribution of globule size (µm) in different pediatric parenteral nutrition (PN) solutions on different days (0, 1, 3, 7) and temperatures (RT, 4ºC). RT: room temperature. *: p.adj < 0.05; **: p.adj < 0.001; ***: p.adj < 0.0001; ****: p.adj < 0.00001.

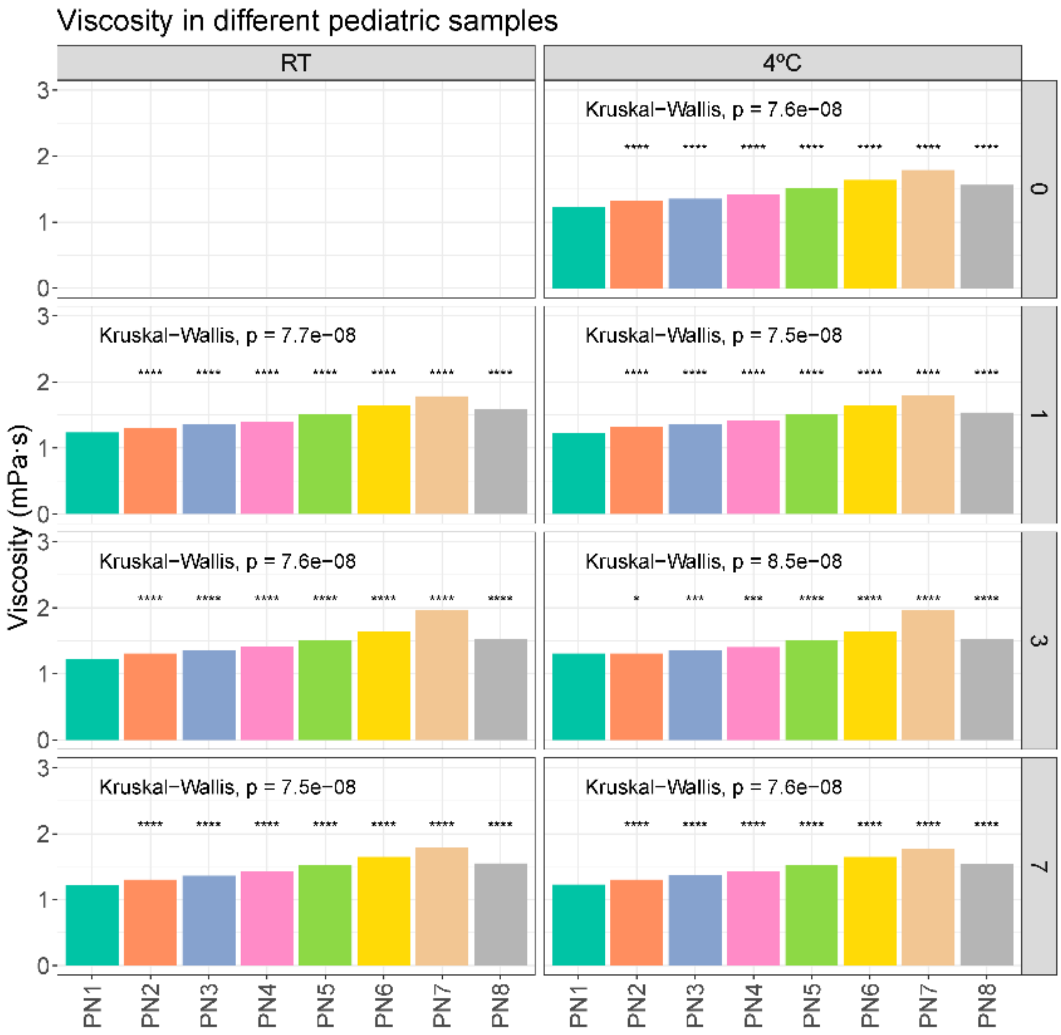

Figure S3. Viscosity (mPa·s) evolution in different pediatric parenteral nutrition solutions on different days (0, 1, 3, 7) and temperatures (RT, 4ºC.). RT: room temperature. Each measurement is the result of the average of 6 determinations for viscosity. *: p.adj < 0.05; **: p.adj < 0.001; ***: p.adj < 0.0001; ****: p.adj < 0.00001.
